# In Vivo Evaluation of 3D-Printed Polycaprolactone Scaffold Implantation Combined with β-TCP Powder for Alveolar Bone Augmentation in a Beagle Defect Model

**DOI:** 10.3390/ma11020238

**Published:** 2018-02-04

**Authors:** Su A. Park, Hyo-Jung Lee, Keun-Suh Kim, Sang Jin Lee, Jung-Tae Lee, Sung-Yeol Kim, Na-Hee Chang, Shin-Young Park

**Affiliations:** 1 Department of Nature-Inspired Nanoconvergence Systems, Korea Institute of Machinery and Materials, Daejeon 34103, Korea; psa@kimm.re.kr (S.A.P.); leesangjin@khu.ac.kr (S.J.L.); 2 Department of Periodontology, Section of Dentistry, Seoul National University Bundang Hospital, Seongnam-si 13620, Korea; periolee@gmail.com (H.-J.L.); alienhd@naver.com (K.-S.K.); icarusecoy@gmail.com (S.-Y.K.); r0387@snubh.org (N.-H.C.); 3 Department of Periodontology, Dankook University, Yongin-si 16890, Korea; jungtae1308@hanmail.net

**Keywords:** 3D printing, polycaprolactone, beta tricalcium phosphate, dentistry

## Abstract

Insufficient bone volume is one of the major challenges encountered by dentists after dental implant placement. This study aimed to evaluate the efficacy of a customized three-dimensional polycaprolactone (3D PCL) scaffold implant fabricated with a 3D bio-printing system to facilitate rapid alveolar bone regeneration. Saddle-type bone defects were surgically created on the healed site after extracting premolars from the mandibles of four beagle dogs. The defects were radiologically examined using computed tomography for designing a customized 3D PCL scaffold block to fit the defect site. After fabricating 3D PCL scaffolds using rapid prototyping, the scaffolds were implanted into the alveolar bone defects along with β-tricalcium phosphate powder. In vivo analysis showed that the PCL blocks maintained the physical space and bone conductivity around the defects. In addition, no inflammatory infiltrates were observed around the scaffolds. However, new bone formation occurred adjacent to the scaffolds, rather than directly in contact with them. More new bone was observed around PCL blocks with 400/1200 lattices than around blocks with 400/400 lattices, but the difference was not significant. These results indicated the potential of 3D-printed porous PCL scaffolds to promote alveolar bone regeneration for defect healing in dentistry.

## 1. Introduction

Some dental surgery patients have suffered from loss of teeth combined with extensive alveolar bone defects caused by tumors, trauma, or severe periodontal disease [1,2]. To repair the biological and functional loss of the oral cavity, comprehensive reconstruction procedures, including implantation of multiple dental implants, are frequently performed [3,4]. However, dental implants cannot be placed in severely atrophic jaw bone due to the loss of bone volume, and thus, various regenerative procedures have been developed [5,6].

To induce rapid bone regeneration, particulate mineralized materials are inserted into the bone defect site along with additional materials, such as a barrier membrane to cover and retain the bone graft materials and/or growth factors to promote bone regeneration. Particulate bone grafting materials are easy to use for many types of bone defects, regardless of the defect morphology. However, they collapse easily when external force is applied unless supported by some rigid structures [7]. Therefore, the success of bone regeneration procedures depends on the shape of the bone defects and whether they can firmly support the bone graft materials. In other words, bone regeneration at the grafted site will be more successful with a concave shape, whereas flat bones and extensive defects can be infected easily, resulting in a low success rate [6,8]. To overcome the weakness of particulate bone graft materials, biodegradable three-dimensional (3D) porous scaffolds have been clinically introduced in dentistry [9].

3D porous scaffolds can maintain the physical space necessary for bone regeneration, thus not only preventing the invasion of undesired cells but also anchoring endogenous osteogenic cells to induce cell ingrowth and providing a molecular environment for osteoblastic differentiation [9]. The fabrication of an ideal personalized scaffold with a precise shape and size has recently become possible with 3D bio-printing systems (3DPs) [10,11]. By analyzing the bone defect site using computer-aided drafting/manufacturing (CAD/CAM) after obtaining 3D images for diagnosing bone defects, a personalized 3D scaffold can be designed and manufactured with 3DPs using the obtained data [12,13,14,15]. Synthetic polymers, such as polycaprolactone (PCL), are commonly used for scaffold fabrication because of their thermoplastic characteristics [16,17,18,19], and their suitability for constructing scaffolds layer-by-layer through 3DPs [20]. In addition, PCL is a safe material approved by the FDA for use in drug delivery devices and implantation scaffolds [21]. Due to its biodegradability and biocompatibility, PCL can be employed as a bone substitute to reconstruct the alveolar bone in the oral cavity.

The major focus of this study was (i) to determine whether β-tricalcium phosphate (β-TCP) powder remains in place when inserted alongside 3D-printed PCL scaffolds; and (ii) to identify the scaffold porosity most suited for bone regeneration. For this purpose, we fabricated scaffolds with two levels of porosity (400/400 and 400/1200 μm) and evaluated the biological responses to the scaffolds in terms of new bone formation and inflammation using an in vivo beagle defect model. The 3D-printed PCL scaffolds were found to be effective in encouraging new bone formation in dental applications.

## 2. Materials and Methods 

### 2.1. Scaffold Fabrication

PCL and β-TCP were purchased from Sigma Aldrich (St. Louis, MO, USA). The geometrical data was obtained from micro Computed Tomography (CT) images, and the scaffolds were fabricated from a stereolithography (STL) file format. The PCL scaffolds were designed with computer-aided design/computer-aided manufacturing (CAD/CAM) and manufactured using a 3D bio-printing system (laboratory lab-made system in Korea Institute of Machinery and Materials, Daejeon, Korea). The scaffolds were printed using a heating dispenser (u-jin tech. Co. Ltd., Gyeonggido, Korea). The 3D bio-printing system comprised a computer-aided 3-axes stage, pressure controller, and dispenser, which controlled the pressure, feeding speed, polymer melting temperature, strand size, and scaffold shape. PCL pellets were melted in a heating jacket at 120 °C, and the 3D scaffold was plotted layer-by-layer using a nozzle size of 400 μm. The scaffold pattern was designed with an orthogonal orientation between the layers with a strand-to-strand distance of 400 and 1200 μm. The strand thickness/period for the two types of scaffolds created was 400/400 and 400/1200 μm. We implanted the PCL block into the defects and applied β-TCP powder to stimulate the bone growth around the defects. The scaffold size of the defect was 10.0 × 5.0 × 5.0 mm. The experimental specimens were divided into three groups: scaffold containing the chopped PCL blocks with less than 2 × 2 mm size and β-TCP powder (Particle group) as the control, scaffold containing 400/400 lattices with β-TCP powder [Block (400/400) group], and scaffold containing 400/1200 lattices with β-TCP powder [Block (400/1200) group]. 

### 2.2. Scaffold Characterization

The morphology of the scaffolds was observed using SEM (scanning electron microscope; NoVa™ nano SEM 200; FEI Company, Hillsboro, OR, USA). The samples were sputter-coated with platinum under an argon atmosphere using a sputter coater (SCD 0005; BAL-TEC, Los Angeles, CA, USA). SEM images were obtained at an accelerating voltage of 10 kV, and the strand size and period of the scaffolds were measured from the SEM images.

The compressive modulus of the scaffolds was measured using a mechanical testing machine (R&B, Daejeon, Korea). The scaffolds were compressed at a crosshead speed of 1 mm/min, and the compressive modulus was calculated as the slope of the linear portion of the curve.

### 2.3. Animal Experiments

**Figure 1:**
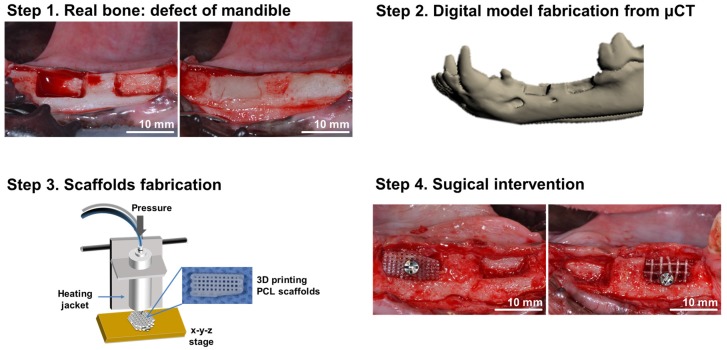
Schematic illustration of the preparation of 3D printed PCL scaffolds for alveolar bone augmentation in a beagle defect model. In the animal, alveolar bone defects were formed and the wax (white) was applied into the defect to maintain the defect volume during the scaffold production. Computed tomography images of the animal were obtained, and a defective mandible model was obtained. The scaffolds were designed using a CAD program and fabricated from PCL using 3D bio-printing techniques. Subsequently, the fabricated scaffolds were implanted into the defects previously formed, and 3 months of healing was allowed.

Four adult male beagle dogs, weighing 12 to 15 kg, were used. The animals had intact dentition with a healthy periodontium. The guidelines regarding the care of animal research subjects were strictly followed, and the research was approved by the Institutional Animal Care and Use Committee of Seoul National University Bundang Hospital, Korea (IACUC No. BA1407-157/033-01). Figure 1 summarizes the surgical procedures.

The beagles were housed in individual cages and fed with a commercial hard food diet (Dog Chow GoldPet, #35520, Cargill Agri Purina, Inc., Pyungtaek, Korea). After fasting for 12 h, the animals were administered a subcutaneous injection of 0.005 mg/kg atropine (Daihan Pharm. Co., Ansan, Korea) in the supine position and anesthetized 15 min later with an intramuscular injection of 5.0 mg/kg zoletil (Zoletil50, Virbac S.A., Carros, France) and 0.2 mg/kg xylazine (Rompun, Bayer Korea, Ansan, Korea). After endotracheal intubation with a 6.5-sized tube, general anesthesia was maintained with 2.2% enflurane (JW Pharmaceutical, Hwasung, Korea), and the oxygen level was maintained at 3.0 L/min. The animals were injected intramuscularly with 30 mg/kg cefazolin (Chongkundang Pharm, Cheonan, Korea) before the surgical procedures. 

The surgical field was scrubbed with a povidone-iodine solution. Both the mandibular second and third premolars were extracted under local anesthesia using 2% lidocaine with 1:100,000 epinephrine (Yuhan Co., Ltd., Seoul, Korea), and a 3-month healing period was allowed. Crestal and vertical incisions were made on the buccal gingiva, and mucoperiosteal flaps in the extracted second and third premolar areas were raised. A standardized rectangular bone defect of 10.0 × 5.0 × 5.0 mm in size [22] was created on the alveolar ridge using a surgical bur. Each bony defect was filled with sterilized utility wax to maintain the defect shape until regenerative therapy. Two days after surgery, the animals were examined with CT under general anesthesia. 

The scaffold design was converted to the STL file format using CAD/CAM. The geometric data was extracted from the STL files, and the scaffolds were printed using a pneumatic dispenser. 

After 2 weeks, surgical re-entry was performed to implant the fabricated 3D scaffolds. The utility wax was removed from the defects, and the scaffolds were installed. The 3D scaffolds were fixed with a fixation screw, and all defect sites were covered with a resorbable membrane (FormaAid^®^ Collagen membrane, Maxigen Biotech Inc., New Taipei city, Taiwan). 

All surgical sites underwent primary closure using polyglactin 4-0 (Vicryl, Ethicon, Menlo Park, CA, USA). Postoperatively, 1.0 mL of dexamethasone-21-isonicotinate (Voren, Boehringer Ingelheim Korea Ltd., Seoul, Korea) was injected once, and 1.0 mL/10.0 kg clemizole penicillin G and sodium penicillin G (Antipen-SM, WooGene B&G Ltd., Seoul, Korea) were injected thrice every second day.

Eight weeks later, two beagles were sacrificed each time through formalin perfusion to evaluate the extent of bone remodeling. Block sections including the grafted sites were harvested and fixed in 70% ethanol at low temperature until micro CT scans were performed.

### 2.4. Micro CT Examination

MicroCT (SkyScan 1173, Skyscan, Kontich, Belgium) scans were performed to evaluate the 3D view of bone remodeling in the harvested bone sections. Digital micro-radiographic images were acquired at 130 kVp and 60 μA using 1.0-mm aluminum filtration. The samples were exposed to radiation at a speed of 500 ms on each rotation of 0.3°, and the pixel size was 19.89 μm. We used amounts of newly formed bone as the outcome measure of this study. For this purpose, newly formed bone areas were measured by histomorphometric analysis and newly formed bone volumes were measured through micro-CT analysis.

### 2.5. Histology and Histomorphometric Analysis

After micro CT scanning, the sections were fixed with 10% buffered neutral formalin (Sigma Aldrich Co. LLC, St. Louis, MO, USA) for 2 weeks and decalcified in formic acid (Shadon TBD-1, Thermo Fisher Scientific Inc., Kalamazoo, MI, USA), followed by a water rinse. The specimens were then decalcified in a tissue processor (Shadon Citadel 2000, Thermo Fisher Scientific Inc., Kalamazoo, MI, USA) and embedded in paraffin with an embedding center (Shadon Histocentre 3, Thermo Fisher Scientific Inc., Kalamazoo, MI, USA). Serial sections of 3.0 μm in thickness were cut using a microtome (Shadon Finesse 325, Thermo Fisher Scientific Inc., Kalamazoo, MI, USA), and each specimen was stained with hematoxylin and eosin.

For histomorphometric analysis, the specimens were processed using the same protocols reported elsewhere [16]. The images were also examined under the same equipment and analyzed using the same software reported in the previous study. The level of new bone formation and the lamellar bone/woven bone ratio were evaluated.

### 2.6. Statistical Analysis

Statistical analysis was performed using commercially available software (STATA/SE14 software, Stata Corp, College Station, TX, USA). Differences between groups were analyzed using the Kruskal–Wallis test. A *p*-value of <0.05 was considered statistically significant. 

## 3. Results

Figure 1 shows the specific procedures followed in this study. Alveolar bone defects were created, and 3D images of the defects were obtained using computed tomography (CT). PCL scaffolds were designed with CAD/CAM and fabricated using 3DPs. For in vivo experiments, the PCL scaffolds were implanted into the defects.

**Figure 2:**
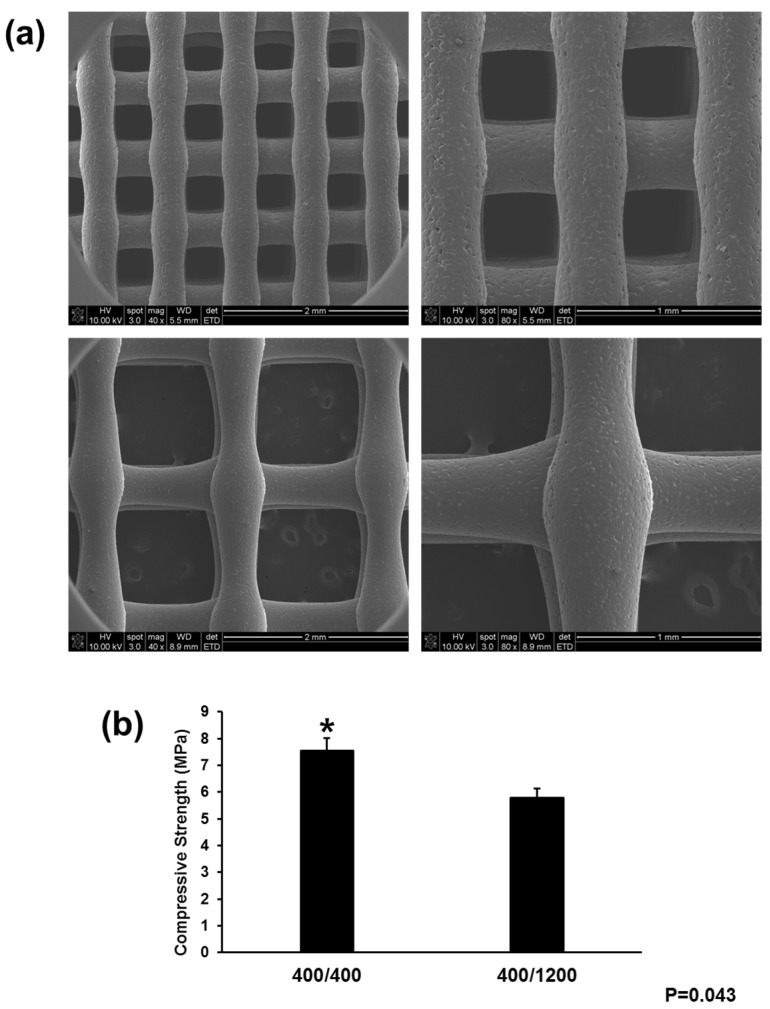
Characterization of scaffold using SEM analysis and measurement of compressive strength. (**a**) SEM images of PCL scaffolds (upper: 400/400 scaffold, lower: 400/1200 scaffold) (original magnification: **Left**, ×40; **Right**, ×80); (**b**) Compression test for the PCL blocks. The ultimate strength of the 400/400 and 400/1200 scaffolds was 7.55 ± 047 MPa and 5.79 ± 0.35 MPa, respectively. “*” indicates statistically significant differences between groups (*p* = 0.043).

To evaluate the shape fidelity and morphology, the 3D-printed scaffolds were observed using scanning electron microscopy (SEM) (Figure 2a). SEM images confirmed that the designed scaffolds had a well-defined and interconnected pore structure consisting of a 3D interconnected structure with micro-sized struts. The diameter of the strands was fixed to 400 μm with a strand-to-strand distance of 400 and 1200 μm. Figure 2b presents the mechanical properties of each scaffold. The compressive strength of the 400/400 scaffolds was higher than that of the 400/1200 scaffolds. Although the 400/1200 scaffolds were slightly weaker, the larger interconnected pore size was better able to promote cell proliferation and osteogenic differentiation for applications in dental surgery and the dental prosthetic field.

**Figure 3:**
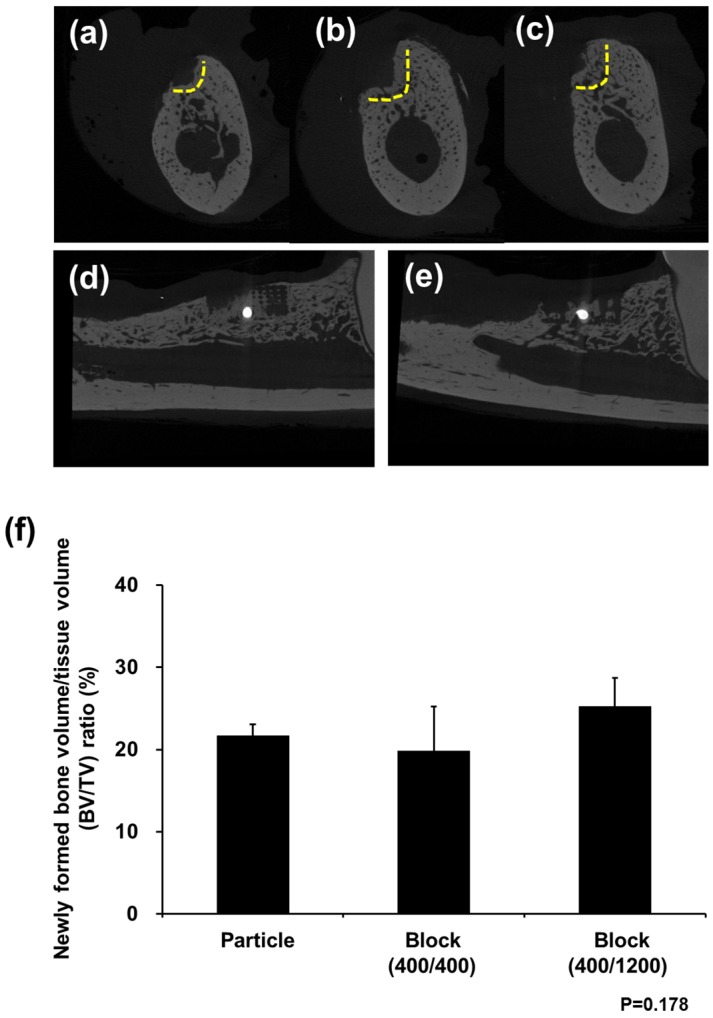
Micro CT images after in vivo experiments. New bone formation was observed above the bottom of the defects (yellow line) in all groups: (**a**) Particle group; (**b**) Block 400/400 group; and (**c**) Block 400/1200 group. The PCL block was radiolucent in micro CT images, and new bone formation between lattices was observed in both the (**d**) Block 400/400 and (**e**) Block 400/1200 groups; (**f**) Bone volume ratio (%) was not significantly different between the groups.

As shown in Figure 3 based on in vivo analysis at 8 weeks after PCL block implantation, each group showed an uneventful healing course. Micro CT analysis revealed the highest new bone formation rate in the Block (400/1200) group, whereas the lowest bone regeneration was found in the Particle group. In addition, the Block groups showed new bone formation through the grid within the PCL block. On the other hand, the new bone showed low radiopacity compared with the native bone. Although the Block (400/1200) group had a larger amount of bone formation than the Block (400/400) group, there was no significant difference (*p* = 0.178).

**Figure 4:**
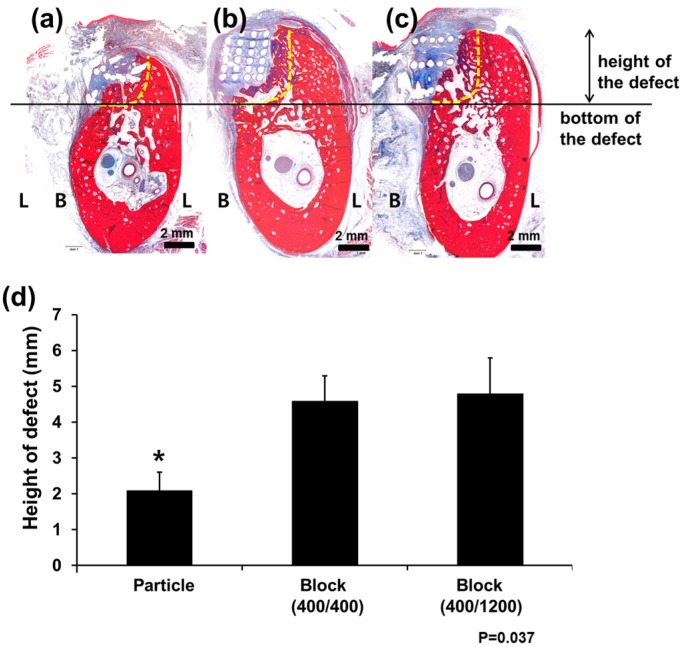
Histological examination of bone regeneration after PCL scaffold implantation. The (**a**) Particle group; (**b**) Block 400/400 group; and (**c**) Block 400/1200 group; original magnification ×1.25. New bone formation was observed above the bottom of the defects (horizontal line) in all groups. The vertical dimensions of the defects were well-preserved in the (**b**) Block 400/400 and (**c**) Block 400/1200 groups, and (**d**) the difference was statistically significant. “*” indicates statistically significant differences between groups (*p* = 0.017). L, lingual side; B, buccal side.

**Figure 5:**
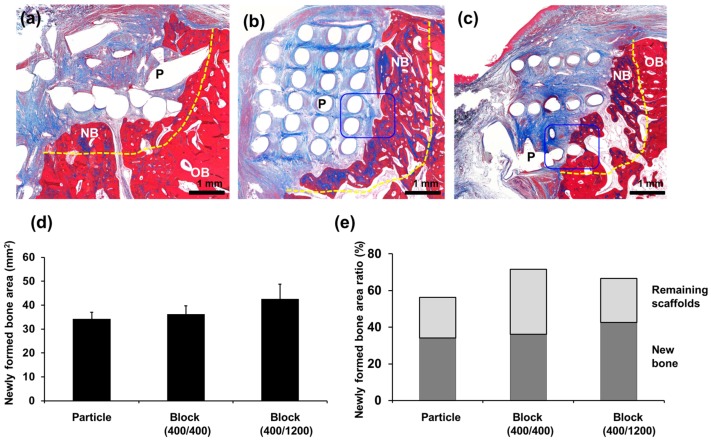
Histology examination of bone regeneration after PCL scaffold implantation. New bone formation was observed above the bottom of the defects (yellow line) in all groups: the (**a**) Particle group; (**b**) Block 400/400 group; and (**c**) Block 400/1200 group; original magnification ×4. Histomorphometric analysis showed no significant differences between groups except that the Block 400/1200 group showed larger amounts of newly formed bone than the other groups; (**d**,**e**) The specimens were decalcified and stained with Masson’s trichrome. B, new bone; OB, old bone; P, PCL scaffold.

Histologically, the pattern of new bone formation was similar to that observed with micro CT examination (Figure 4 and Figure 5). Measuring the heights of the defects, the PCL Block groups showed a well-maintained vertical height for the defects, whereas bone was vertically resorbed in the PCL Particle group (*p* = 0.037). Most of the scaffold was intact in the defects, and >60% of the defects were filled with newly formed bone and scaffolds in the PCL Block groups.

**Figure 6:**
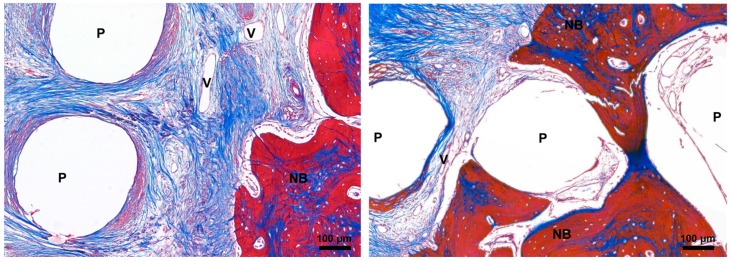
Magnification (×40) of newly formed bone and PCL scaffolds. The specimens were decalcified and stained with Masson’s trichrome. NB, new bone; P, PCL scaffolds; V, vessel.

New bone formation without penetration into the grid space was observed around the scaffolds in the Block 400/400 group, whereas newly formed bone was observed in the grid space in the Block 400/1200 group. Direct contact was not observed between the PCL scaffolds and the newly formed bone, although some fibrous tissue was observed in the grid space. The infiltration of inflammatory cells was not observed (Figure 6).

## 4. Discussion

Recently, a variety of designs has become available for tissue-engineered scaffolds using PCL polymer due to the plasticity and biocompatibility of the materials [12]. In this study, PCL was used to fabricate latticed blocks for dental applications to encourage bone regeneration into the scaffolds. The PCL scaffold successfully maintained the physical space at the bone defect site and facilitated the regeneration of healthy bone with no inflammatory or infectious reactions after surgery. Overall, the 3D bio-printing-based PCL scaffolds demonstrated a potential for bone healing applications in dentistry.

We demonstrated that PCL block scaffolds are advantageous to preserving the vertical dimensions of bone defects for dental applications. After the extraction of a natural tooth, alveolar bone resorption occurs even if dental implants are placed immediately [23]. To minimize the loss of the vertical dimension, dentists typically perform a socket preservation procedure by immediately filling the extraction socket with bone graft materials when bone volume loss is expected. Nevertheless, the vertical dimension required for the placement of implants is frequently not secured as observed with the Particle group and negative control in this study. Extensive bone loss was observed in negative control group and no vertical wall of the defects was observed (please see Figure S1 in supplementary information). Vertical dimension loss in the defects was reduced in this study using PCL blocks to maintain the height of the alveolar bone. In contrast, the Particle group did not overcome this deficiency.

The preservation of the vertical dimension in bone defects was primarily due to the structural stability of the PCL scaffolds, which were barely absorbed after 8 weeks and stably supported the defects. In addition, because the PCL scaffolds did not generate by-products when decomposed, inflammatory infiltrates were not observed around the scaffolds. However, the scaffolds were not replaced by bone and intimate bone contact was also not observed. Separation was observed between the bone and the materials, and fibrous encapsulation between the materials was visible, even though β-TCP had been applied to promote new bone formation on the PCL scaffolds. In dentistry, bone substitutes without direct bone contact are unfavorable for the long-term stability of newly formed bone because of the poor bone quality [24,25,26]. There have been several attempts to improve bone apposition on the surface of PCL, such as applying β-TCP coating on the PCL or using hydroxyapatite-coated PCL scaffolds [10,20,27,28]. Other attempts have been made to improve PCL absorption, such as increasing the hydrophilic properties or the porosity of the PCL and changing the composition of PCL (PCL/Poly Lactic-co-Glycolic Acid) [17,29,30]. However, given the results of this study, PCL scaffolds appear to be more suited to being temporary bone graft materials than permanently submerged materials.

New bone formation was observed around the scaffold in both groups and the amount of new bone was not significantly different. Interestingly, the pattern of the new bone formation was different between scaffolds. New bone formation scarcely occurred between the grids in the scaffold with a narrow lattice group. Rather, new bone formation was observed around the scaffolds. However, in the scaffold with a wide lattice group, new bone formation was observed between the grids. As a result, the PCL with a wide lattice had an advantage in the amount of new bone formation. Although mechanical test showed that PCL scaffolds with wide lattice were disadvantageous than those with narrow lattice, scaffolds with wide lattices were also stable after 8 weeks and able to resist the oral external force. In addition, the large interconnected pore size can promote cell proliferation and osteogenic differentiation for new bone formation.

In vivo experiments revealed a difference in volume between the 3D scaffold blocks fabricated with the 3D bio-printer and the actual defects. The 3D-printed PCL scaffold blocks were larger than the actual defect size and required some trimming before transplantation into the defect site. These results may be attributed to an error caused by distortions between the CT images and the actual structure that developed in the process of creating a 3D image based on the obtained data [31,32]. Further research will be needed to compensate for the size differences resulting from the common output errors and the differences between the CAD/CAM design and the final image utilized in the actual output for 3D bio-printing.

This study had several limitations in addition to the sample size. Because the study was intended to be a pilot study for the dental application of PCL scaffolds, the sample size was small, and the results failed to achieve statistical significance. 

## 5. Conclusions

In summary, PCL block scaffolds designed for bone regeneration were biocompatible and effective at maintaining space in areas with dental defects. The lattice size did not significantly affect bone regeneration. However, the rigid structure of the PCL block was more helpful in supporting the vertical dimensions of the defects and facilitating bone regeneration.

## Supplementary Materials

The following are available online at http://www.mdpi.com/1996-1944/11/2/238/s1, Figure S1: Histologic image of negative control (original magnification X1.25). Sham surgery was performed and no graft material was applied. Due to extensive bone loss, border between native bone and newly formed bone was not distinguished.
