# Supplementary material for: In Vivo Evaluation of 3D-Printed Polycaprolactone Scaffold Implantation Combined with β-TCP Powder for Alveolar Bone Augmentation in a Beagle Defect Model
Source: Materials (Basel). 2018 Feb 4;11(2):238. doi: 10.3390/ma11020238 (PMC5848935; doi:10.3390/ma11020238)
Supplement: Supplementary file 1 [file materials-11-00238-s001.pdf]

materials

*Supplementary Information*

# In Vivo Evaluation of 3D-Printed Polycaprolactone Scaffold Implantation Combined with β-TCP Powder for Alveolar Bone Augmentation in a Beagle Defect Model

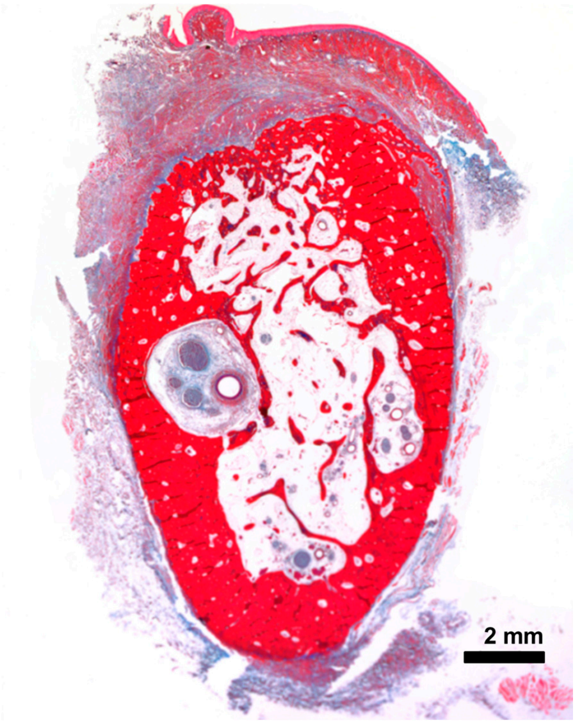


**Figure S1.** Histologic image of negative control (original magnification X1.25). Sham surgery was performed and no graft material was applied. Due to extensive bone loss, border between native bone and newly formed bone was not distinguished.
